# The influence of allelic variants of the Vrn-A1 gene on the duration of the vegetation period in Triticum dicoccoides

**DOI:** 10.18699/vjgb-25-132

**Published:** 2025-12

**Authors:** G.Yu. Chepurnov, Z. Chen, A.G. Blinov, V.M. Efimov, N.P. Goncharov

**Affiliations:** Siberian Research Institute of Plant Production and Breeding – Branch of the Institute of Cytology and Genetics of the Siberian Branch of the Russian Academy of Sciences, Krasnoobsk, Novosibirsk region, Russia Institute of Cytology and Genetics of the Siberian Branch of the Russian Academy of Sciences, Novosibirsk, Russia; Institute of Cytology and Genetics of the Siberian Branch of the Russian Academy of Sciences, Novosibirsk, Russia Novosibirsk State University, Novosibirsk, Russia; Institute of Cytology and Genetics of the Siberian Branch of the Russian Academy of Sciences, Novosibirsk, Russia; Institute of Cytology and Genetics of the Siberian Branch of the Russian Academy of Sciences, Novosibirsk, Russia Novosibirsk State University, Novosibirsk, Russia; Institute of Cytology and Genetics of the Siberian Branch of the Russian Academy of Sciences, Novosibirsk, RussiaNovosibirsk State University, Novosibirsk, Russia

**Keywords:** wild emmer, Triticum dicoccoides, spring growth habit, Vrn-A1 gene, alleles, duration of vegetation period, 2B-PLS analysis, дикая полба, Triticum dicoccoides, яровой тип развития, ген Vrn-A1, аллели, длина вегетационного периода, 2B-PLS анализ

## Abstract

The duration of the vegetation period (DVP) is an important agronomic trait in cereal. Тhe main influence on it in wheat is exerted by Vrn genes, which determine the growth habit (spring vs. winter) and DVP. In the present study, 137 wild emmer Triticum dicoccoides (Körn. ex Aschers. et Graebn.) Schweinf. accessions were evaluated according to the growth habit trait, among which 39 spring ones were identified. The nucleotide sequences of the promoter region of the Vrn-A1 gene were established in the spring accessions by sequencing. Five allelic variants of Vrn-A1 genes previously found in T. dicoccoides were identified, namely Vrn-A1b.1, Vrn-A1b.2, Vrn-A1b.4, Vrn-A1d, Vrn-A1u. Three spring accessions PI355457, PI190919, PI560817 simultaneously contained two alleles of the Vrn-A1 gene: Vrn-A1d and previously undescribed functional allelic variant designated by the authors as Vrn-A1b.8. The promoter region of this allele had several deletions relative to the intact variant. One of such deletions covered 8 bp of the VRN box. In a single experiment, under controlled greenhouse conditions, the relationship between the allelic variants of the Vrn-A1 gene and the duration of the vegetation period of the T. dicoccoides’ spring accessions was studied using the 2B-PLS (Two-Block Partial Least Squares) analysis. The correlation coefficient (r) between these traits was 0.534. The correlation coefficient between the duration of the vegetation period of wild emmer plants and the regions of origin of the studied accessions was also calculated (r = 0.478). It was shown that accessions with identical alleles of the Vrn-A1 gene and originating from the same region can differ significantly from each other in the duration of the vegetation period. The presence of phenotypic differences with the same allelic composition of the Vrn-A1 gene indicates the contribution of other hereditary factors localized in the genomes of these accessions, which determines their value as new donors of genetic resources that contribute to the expansion of the biodiversity of common and durum wheat commercial cultivars.

## Abstract (ru)

Длина (продолжительность) вегетационного периода является одним из ключевых хозяйственно важных признаков зерновых культур. У пшениц основное влияние на него оказывают гены Vrn, контролирующие тип (яровость vs. озимость) и скорость развития растений. В настоящей работе по признаку «тип развития» изучено 137 образцов дикой полбы Triticum dicoccoides (Körn. ex Aschers. et Graebn.) Schweinf., среди которых выявлено 39 яровых. Методом секвенирования у яровых образцов установлены нуклеотидные последовательности промоторной области гена Vrn-A1. Идентифицировано пять аллельных вариантов: Vrn-A1b.1, Vrn-A1b.2, Vrn-A1b.4, Vrn-A1d, Vrn-A1u. Три яровых образца, PI355457, PI190919 и PI560817 T. dicoccoides, содержали одновременно два аллеля гена Vrn-A1: Vrn-A1d и не описанный ранее аллельный вариант, обозначенный авторами как Vrn-A1b.8. Промоторная область этого аллеля имела несколько делеций относительно интактного варианта, одна из которых захватывала 8 п. н. VRN-бокса. В контролируемых условиях теплицы у образцов была определена длина вегетационного периода и методом 2B-PLS анализа (two-block partial least squares analysis) оценена связь между аллельными вариантами гена Vrn-A1 и длиной вегетационного периода яровых образцов T. dicoccoides. Коэффициент корреляции (r) между этими признаками составил 0.534. Был рассчитан также коэффициент корреляции между длиной вегетационного периода растений и регионами происхождения образцов (r = 0.478). Показано, что образцы, имеющие одинаковые аллели гена Vrn-A1 и происходящие из одного региона, могут значительно отличаться между собой по длине вегетационного периода. Наличие фенотипических различий при одинаковом аллельном составе гена Vrn-A1 указывает на вклад иных наследственных факторов, локализованных в геномах этих образцов, что обусловливает их ценность в качестве новых источников генетических ресурсов, способствующих расширению биоразнообразия сортов возделываемых видов пшениц.

## Introduction

Wild emmer Triticum dicoccoides (Körn. ex Aschers. et Graebn.) Schweinf. is one of the hypothetic ancestors of cultivated tetraploid (2n = 4x = 28) wheat (Rivera et al., 2025). Its range covers the Fertile Crescent (Southwest Asia) and extends from Israel, Jordan, Lebanon, Syria, southern Turkey and northern Iraq to southwestern Iran (Özkan et al., 2011; Lack, van Slageren, 2020), where emmer T. dicoccum Schrank ex Schübl. was domesticated (Novoselskaya-Dragovich et al., 2025). Due to this extensive areal, T. dicoccoides retains polymorphism and has significant potential to improve modern cultivated wheat species (Kato et al., 1997; Nevo, 2001; Dong et al., 2010; Feng et al., 2017). Allelic variants of T. dicoccoides genes determining agronomically important traits have often been used to improve the resistance of durum and bread wheat plants to various diseases such as ear fusarium (Soresi et al., 2017, 2021), yellow rust (Sela et al., 2014; Zhang H. et al., 2016), powdery mildew (Xue et al., 2012; Ouyang et al., 2014; Liang et al., 2015; Saidou et al., 2015; Qiu et al., 2021), and others. In addition to the introgression of immunity-related genes, wild emmer is widely used to improve other traits in cultivated wheat species, such as increased adaptability due to the transfer of dominant Vrn (response to vernalization) genes and their alleles responsible for the formation of the growth habit (spring vs. winter) and the duration of the vegetation period (Strejčková et al., 2023).

The growth habit is the most important trait that determines wide adaptability of wheat plants to various climatic conditions (Law, Worland, 1997). Winter-type wheat requires prolonged exposure to low positive temperatures (typically ≥50 days of vernalization) for transition from vegetative to reproductive development (Kiss et al., 2025). This mechanism causes a delay in the vegetative phase of plants, preventing damage to floral meristems by low temperatures. Spring wheat delays the transition from vegetative to reproductive development during a single growing season without vernalization (Distelfeld et al., 2009a). It has been more than once shown that the Vrn genes, which control the growth and development characteristics (duration of ontogenesis) of wheat plants, determine not only the growth habit (spring/winter), but also the duration of development phases (Efremova, Chumanova, 2023), i. e. they control the duration of the life cycle from germination to ripening and, as a result, affect early flowering and yield (Flood, Halloran, 1986; Goncharov, 1998; Distelfeld et al., 2009a; Kamran et al., 2014; Shcherban et al., 2015a; Afshari-Behbahanizadeh et al., 2024; Plotnikov et al., 2024; etc.). In addition to these genes, the duration of the vegetation period in wheat is also affected by other genes, such as Ppd (response to photoperiod), which determine the sensitivity of plants to photoperiod, and Eps (earliness per se), which determine the earliness without the influence of external signals (Distelfeld et al., 2009a; Kamran et al., 2014; Würschum et al., 2018). It is noted that the Vrn gene system accounts for up to 75 % of variability for this trait, while the other two systems – for about 25 % (Stelmakh, 1998). The significant influence of Vrn genes on phenology (particularly flowering time regulation) has motivated extensive research into these loci. By now, studies have characterized these genes’ genomic structure and chromosomal localization, while also elucidating their interactions with other genes controlling developmental timing (Yan et al., 2003, 2004b, 2006; Fu et al., 2005; Distelfeld et al., 2009b; Chen A., Dubcovsky, 2012; Shcherban et al., 2012a, b, 2013, 2015a; Chen F. et al., 2013; Kippes et al., 2014–2016; Shcherban, Salina, 2017).

Mutations of three genes, Vrn-1, Vrn-2 and Vrn-3, cause the spring growth habit in many species of the genus Triticum L. (Goncharov, 2004a, b; Yan et al., 2004a, b, 2006; Golovnina et al., 2010; Shcherban, Salina, 2017). In common wheat (Triticum aestivum L.) (Kippes et al., 2014, 2015) and T. sphaerococcum Perc. (Goncharov, Shitova, 1999), the fourth Vrn gene, Vrn-D4, has been described. The expression of Vrn-1 serves as the primary molecular trigger initiating the inflorescence development cascade (Yan et al., 2003; Trevaskis et al., 2007). The Vrn-1 gene encodes MADS-box transcription factors (Yan et al., 2004a; Trevaskis et al., 2007), which reduce the expression of Vrn-2 genes and induce the expression of Vrn-3 genes, which function as florigen (Dubcinsky et al., 2006; Yan et al., 2006; Hemming et al., 2008). It has been shown that the spring growth habit in hexaploid (2n = 6x = 42) wheat can be determined by mutations in the Vrn-1, Vrn-D4 and Vrn-3 genes, which cause their expression without low temperature (vernalization) (Yan et al., 2003, 2004b, 2006; Fu et al., 2005; Chen A., Dubtsovsky, 2012; Shcherban et al., 2012a, b, 2013, 2015a; Kippes et al., 2014, 2015; Shcherban, Salina, 2017), or due to a decrease in the number of zinc finger domains and CCTs that form the Vrn-2 codes, or form a cyclic composition of domain structures (Distelfeld et al., 2009b; Chen F. et al., 2013; Kippes et al., 2016).

The spring growth habit in T. dicoccoides is inherited in a dominant manner (Goncharov, 1998). In this species, allelic variants of the gene determining the spring growth habit are described only in the VRN-1 locus (Yan et al., 2004a; Shcherban et al., 2015b; Konopatskaya et al., 2016; Muterko et al., 2016; etc.). To date, seven such alleles are known, four of which contain deletions of different lengths in the promoter region (Vrn-A1b.2, Vrn-A1b.7, Vrn-A1f and Vrn-A1d ); two alleles have the structure of these elements in the same region (Vrn-A1a.3) and a deletion in the first intron (Vrn-A1c); one allele differs from the intact sequence by 29 nucleotide substitutions, one deletion and one SNP insertion in the promoter region (Vrn-B1dic) (Yan et al., 2004a; Shcherban et al., 2015b; Konopatskaya et al., 2016; Muterko et al., 2016).

All of the above-mentioned allelic variants of Vrn-1 genes were previously detected in a study of 92 spring and winter accessions of T. dicoccoides (Yan et al., 2004a; Shcherban et al., 2015b; Konopatskaia et al., 2016; Muterko et al., 2016). However, these studies cover only a portion of the wild emmer accessions available in collections. According to the GRIN NPGS report, based on the results of 2001 trials, 792 T. dicoccoides accessions were sown at the USDA research station in Idaho. 292 of them were classified as spring or facultative forms (URL: https://npgsweb.ars-grin. gov/gringlobal/method?id=491608, accessed April 2, 2025). However, unlike bread wheat, the studies published to date do not provide information on the effect of the identified allelic variants of Vrn-1 genes on the change in the duration of the growing season of spring T. dicoccoides plants

The present study has two main objectives: (i) sequencing and analysis of the promoter region of the Vrn-A1 gene, including VRN-box and GArG-box, in 39 previously unstudied spring accessions of T. dicoccoides, (ii) assessment of the associative relationship between the allelic variants of the Vrn-A1 gene and the duration of the growing season in spring accessions of T. dicoccoides under controlled conditions.

## Materials and methods

**Fig. 1.**
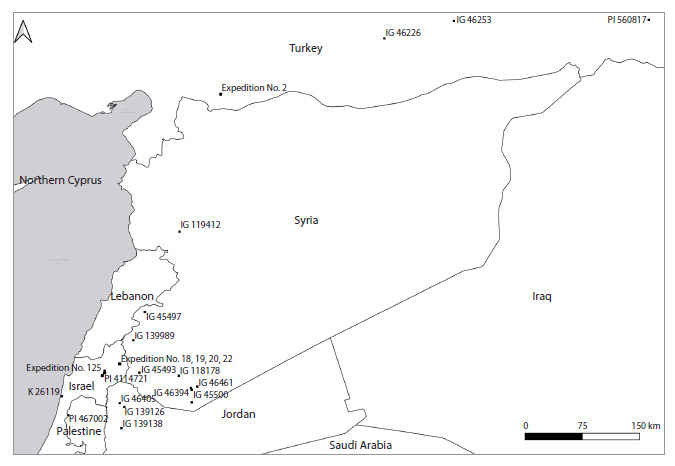
Map of the collection sites of T. dicoccoides accessions studied in this work.

Plant material, growing conditions, assessment of the growth habit and duration of the vegetation period. The plant material for the study was 137 T. dicoccoides accessions of various ecological and geographical origins, of which 39 accessions with a spring growth habit were identified and taken for further study (Table 1, Fig. 1). Progeny seeds were obtained from each accession to assess the growth habit (spring vs. winter) and heading time. The plants were planted as 5-day-old seedlings (10 per accession) in a hydroponic greenhouse of the Institute of Cytology and Genetics SB RAS without preliminary vernalization. The plants were grown at a temperature of 23–25 °C, under long-day (16 h) conditions, at standard humidity. The number of days from planting to heading was recorded for each plant individually. Based on the data obtained, the average value of this feature for each accession was estimated.

**Table 1.**
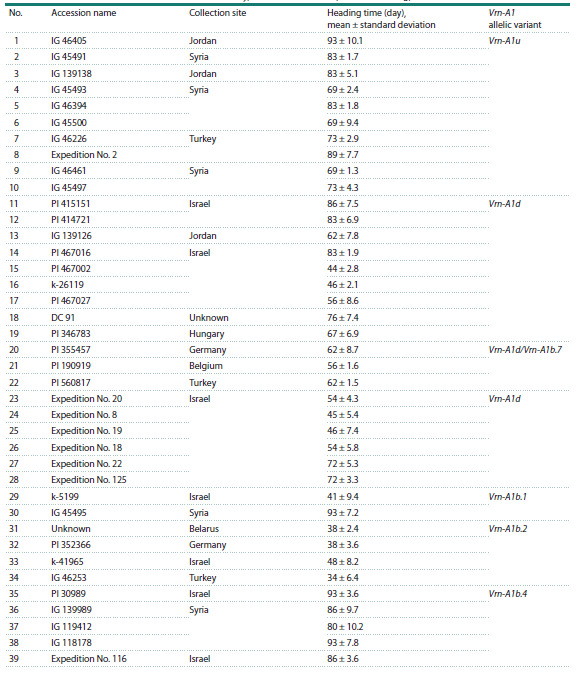
Triticum dicoccoides accessions used in the study, their collection sites, time to heading, and Vrn-A1 allelic variants

Total DNA isolation, PCR amplification, and nucleotide sequence analysis of the Vrn-A1 gene promoter. Total DNA was isolated using the DNeasy Plant Mini Kit (QIAGEN, Hilden, Germany) according to the manufacturer’s protocol 50–100 mg of freshly collected leaves from each sample were used for DNA extraction. The quantity and quality of the isolated DNA were determined using a NanoDrop2000 spectrophotometer (Thermo Scientific, Waltham, USA) and electrophoretic separation in 1 % agarose gel containing ethidium bromide (0.5 mg/ml) in 1xTAE. Polymerase chain reactions (PCR) were performed in 20-μl reactions containing 10 mM Tris-HCl (pH 8.9), 1 mM (NH4)2SO4, 4 mM MgCl2, 200 μM of each dNTP, 0.5 μM of each primer, 1 unit of Taq DNA polymerase, and 0.1 μg of genomic DNA. Primers VRN1AF (5′-GAAAGGAAAAATTCTGCTCG-3′) and VRN1-INT1R (5′-GCAGGAAATCGAAATCGAAG-3′) were used to probe the target region of the promoter region. The primers amplified a 688 bp fragment (in the case of the intact allelic variant) from position –439 bp upstream of the start codon to 246 bp upstream of the start codon. The PCR program included an initial denaturation step for 5 min at 94 °C and 33 amplification cycles consisting of 30 sec of denaturation at 94 °C, 40 sec of annealing at 52 °C and 1 min of elongation at 72 °C. PCR products were separated by agarose gel electrophoresis and purified using the QIAquick Gel Extraction Kit (QIAGEN). PCR products were cloned into the pAL2-T vector using the Quick-TA kit protocol (Eurogen, Moscow, Russia). Sequencing reactions were performed using 200 ng of product and the BigDye Terminator v3.1 sequencing kit (Thermo Scientific, USA) on an ABI 3130XL genetic analyzer (Applied Biosystems, Waltham, MA, USA) at the Genomics Center of the Siberian Branch of the Russian Academy of Sciences (URL: http://www.niboch.nsc.ru/doku.php corefacility, accessed April 2, 2025).

Statistical analysis of data. For statistical processing, quantitative and qualitative characteristics of the samples were used. The analysis of allelic variants of the Vrn-A1 gene and their relationship with the heading date (duration of the vegetation period) was carried out taking into account previously published data obtained under identical growing conditions (hydroponic system, 16-hour photoperiod) (Shcherban et al., 2015b; Konopatskaia et al., 2016; Muterko et al., 2016). The data are presented as a “samples–features” matrix. Each object is described by three features: the heading date of the sample (quantitative), the allelic variant of the Vrn-A1 gene (qualitative), and the region of accession collection sites (qualitative). The two-block partial least squares method (2B-PLS) was applied to each pair of blocks (Rohlf, Corti, 2000). Then, the correlations between the obtained bicomponents were calculated. Calculations and visualization of the obtained results were carried out in the software package for statistical analysis Statistica 12.6 (StatSoft).

## Results

**Study of growth habit and duration of the vegetation period in the studied T. dicoccoides accessions**

To study the growth habit (spring/winter type) of plants, 137 accessions of wild emmer T. dicoccoides were planted at a hydroponic greenhouse at the Institute of Cytology and Genetics SB RAS. Among the studied accessions, 12 (IG 45495, IG 45491, IG 46394, PI 414721, IG 45500, PI 355457, PI 560817, k-41965, k-26119, PI 467002, k-5199, PI 352366) had been previously characterized as spring ones (URL: https://npgsweb.ars-grin.gov/gringlobal/method?id=491608; URL: https://www.genesys-pgr.org; URL: https://grs.icarda. org, accessed April 2, 2025). These were used as spring controls. Among the controls, plants of accession IG 45495 (Syria) were the latest heading. Of the 125 previously unstudied accessions, 98 did not do transition to reproductive development and remained at the tillering stage, while the remaining 27 accessions formed spikes no later than the late spring control IG 45495. Considering that all seedlings were planted without vernalization, we classified 98 accessions that failed to head as winter types, and 27 accessions as spring ones. In subsequent studies of the nucleotide sequences of Vrn-A1 gene alleles, only the 27 identified spring accessions and 12 spring controls were studiedFor all spring accessions, the duration of the period from seedling planting to heading (in days) was recorded. The plants exhibited substantial variation in the duration of the vegetation period; the earliest-maturing accession, IG 46253 (Turkey), reached heading in 34 days, while the latest-maturing one, IG 45495, took 93 days (Table 1). The obtained data were used to calculate the correlation between the allelic variants of the Vrn-A1 gene and the duration of the vegetation period in plants.

**Analysis of the nucleotide sequences of the promoter region of the Vrn-A1 gene**

**Fig. 2.**
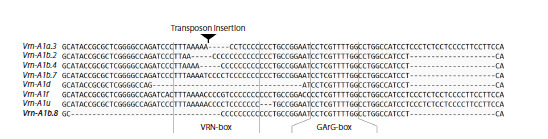
Alignment of Vrn-A1 gene promoter sequences found in 39 spring T. dicoccoides. The newly identified sequence is shown in bold.

The studied T. dicoccoides accessions revealed six distinct variants of the Vrn-A1 gene promoter. Five variants corresponded to alleles previously described in this species. Ten accessions carried the Vrn-A1u allele; two, Vrn-A1b.1; four, Vrn-A1b.2; five, Vrn-A1b.4; and eighteen, Vrn-A1d (Table 1). Among 39 analyzed sequences, none contained additional SNPs or other mutations in the VRN-box and GArG-box regions compared to previously described variants (Fig. 2). Three accessions (PI 355457, PI 190919, PI 560817) were of particular interest as they simultaneously contained two Vrn-A1 gene promoters. One sequence was identical to the Vrn-A1d allelic variant. The other sequence contained three deletions relative to the intact promoter variant: a 32 bp deletion located between –234 bp and –201 bp upstream of the start codon; a 19 bp deletion between –159 bp and –139 bp upstream; and a 1 bp deletion at –138 bp upstream. The deletion located farthest from the start codon encompassed 8 bp of the VRN-box, while the remaining sequence of this site contained a T to C substitution at position –197 bp upstream. This allelic variant, discovered in the present study, was designated as Vrn-A1b.8. All sequences have been deposited in GenBank (accession numbers PV699347–PV699388).

**2B-PLS analysis**

For statistical analysis of the obtained results, we used three data blocks describing the accessions: heading time (quantitative trait), Vrn-A1 gene allelic variants (the number of binary traits equals the number of alleles), and region of origin (the number of binary traits equals the number of origin regions). For each pair of blocks (heading–allele, heading–region of origin, allele–region of origin), we applied the two-block partial least squares method (2B-PLS) (Rohlf, Corti, 2000). The data on Vrn-A1 gene allelic variants and heading times of accessions obtained in the present study were supplemented with similar data from previously published studies where plants were grown under identical conditions (hydroponic greenhouse, 16-hour photoperiod) (Shcherban et al., 2015b; Konopatskaia et al., 2016; Muterko et al., 2016).

**Fig. 3.**
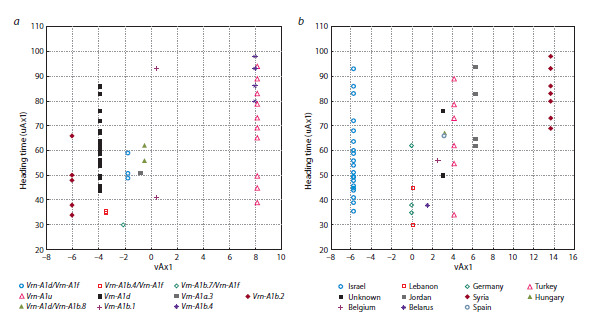
Configuration of samples on the planes of the first bicomponents. a – vAx1 calculated based on allelic variants of the Vrn-A1 gene, b – vAx1 calculated for regional samples.

During the analysis, we also considered information about the geographic origin (collection site) of accessions to evaluate the influence of different Vrn-1 gene alleles on plant heading time, contributing to wheat adaptation to environments. For accessions containing two Vrn-A1 alleles, we treated them as a separate variant, recording the alleles present in the accession as separated by a slash (e. g., Vrn-A1d/Vrn-A1f ). We only considered the first pair of axes (designated as uAx1 and vAx1) showing the highest covariance (Fig. 3). When a block contained only one trait (“heading time”), it constituted the sole (first) bicomponent of that block (uAx1).

When applying 2B-PLS analysis to assess the relationship between the duration of the vegetation period and allelic variants of the Vrn-A1 gene, we derived one axis (uAx1) from the “heading time” trait and another axis (vAx1) from the binary “accession–allele” matrix. The correlation coefficient (r) between the axes was 0.53 (moderate association), p-value = 3.88 × 10–6 (Table 2). No specific allelic variant of the Vrn-A1 gene showed a correlation coefficient with axis uAx1 ≥0.5. The only allele with a close value (0.45) was Vrn- A1b.4, while other allelic variants showed correlations <0.3 with the “heading time” trait. The strongest associations with axis vAx1 were observed for alleles Vrn-A1d (r = –0.61) and Vrn-A1u (r = 0.8).The opposite signs of the correlation coefficients for these two allelic variants suggest their opposing effects on the trait

**Table 2.**
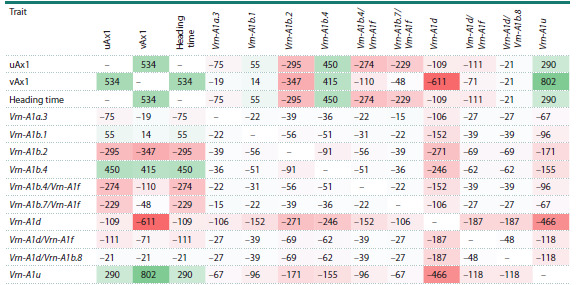
Correlation matrix (×1000) between Vrn-A1 allelic variants and plant heading time Note. Color here and in Table 3: light red, light green – p < 0.001, red, green – p <10–4.

Using the same method, we analyzed the influence of genetic adaptation mechanisms by deriving axis vAx1 from the “accession–origin region” matrix. The second axis (uAx1), as in the previous case, corresponded to the “heading time” trait. The correlation coefficient between the axes showed a weaker association than in the analysis of the relationship between vegetation period duration and Vrn-A1 alleles (r = 0.47, p-value = 4.92 × 10–5) (Table 3). Accessions from Syria showed a correlation coefficient with plant heading time of r = 0.46, while accessions from other regions demonstrated insignificant associations with this trait (r < 0.3). The strongest associations with axis vAx1 were observed for accessions from Israel and Syria (r = –0.864 and r = 0.812, respectively). The difference in signs of the correlation coefficients indicates opposing effects of different genetic adaptation mechanisms on the duration of the vegetation period.

**Table 3.**
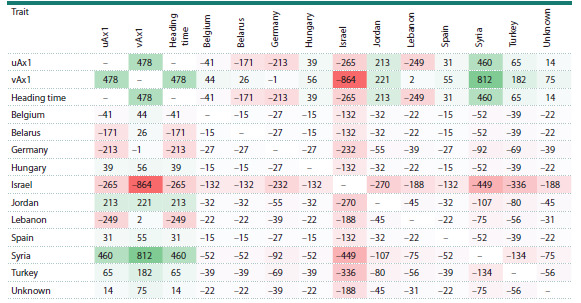
Matrix of relationships (×1000) between the region of origin or habitat of accession and the time of plant heading Note. For Germany, Hungary, Belarus, and Belgium, these indicate not natural habitats, but GenBank deposit locations.

## Discussion

Combinations of allelic variants of Vrn-1 genes significantly influence the agronomically important trait of “duration of the vegetation period” in cultivated wheat species (Flood, Halloran, 1986; Goncharov, 1998; Kato et al., 1998; Distelfeld et al., 2009a; Kamran et al., 2014; Shcherban et al., 2015a; Smolenskaya et al., 2022; Smolenskaya, Goncharov, 2023; Afshari-Behbahanizadeh et al., 2024; Plotnikov et al., 2024; Goncharov et al., 2025). Studying the allelic composition of these genes in wild species and the relationship between different alleles and vegetation period duration is crucial for expanding diversity and enhancing plasticity of cultivated species, as well as their adaptation to specific growing conditions in different regions. Populations of T. dicoccoides contain spring accessions where mutant variants of Vrn-1 genes emerged independently from those in widely cultivated T. aestivum and T. durum Desf. (Shcherban et al., 2015b; Konopatskaia et al., 2016; Muterko et al., 2016). Introgression of wild emmer alleles into cultivated species would allow to expand their polymorphism and enable finer tuning of heading times in commercial cultivars.

In this study, we examined the growth habit (spring vs. winter) in 137 T. dicoccoides accessions and analyzed the promoter region of the Vrn-A1 gene in 39 identified spring accessions. Thirty-six of them contained five allelic variants (Vrn-A1b.1, Vrn-A1b.2, Vrn-A1b.4, Vrn-A1d, Vrn-A1u) previously described in T. dicoccoides (Yan et al., 2004a; Shcherban et al., 2015b; Konopatskaia et al., 2016; Muterko et al., 2016). The presence of the Vrn-A1b.1, Vrn-A1b.2 or Vrn-A1d alleles in wild emmer genomes has been shown to be a determining factor for spring growth habit (Yan et al., 2004a; Shcherban et al., 2015b; Konopatskaia et al., 2016; Muterko et al., 2016). Three of the 39 spring T. dicoccoides accessions simultaneously carried two different Vrn-A1 variants. One allele sequence matched the previously described Vrn-A1d promoter variant, while the other contained deletions differing from known variants. The National Center for Biotechnology Information (NCBI) database contains no nucleotide sequences identical to this allelic variant. The closest match was Vrn-A1b.7, from which our newly identified variant differed by a 32 bp deletion located between –234 bp and –201 bp upstream of the start codon, and a T to C substitution at –197 bp upstream. We designated this novel variant as Vrn-A1b.8. The deletion in the Vrn-A1b.8 promoter encompassed 8 bp of the VRN-box region.

Searching for Vrn-A1 promoter sequences with similar VRN-box deletions revealed the closest match to be the Vrn-A1o allele (Zhang B. et al., 2023), which carries a larger (10 bp) deletion from the 5′ end of VRN-box. The Vrn-A1o allele has been shown to confer spring growth habit in common wheat (Zhang B. et al., 2023). This suggests that Vrn-A1b.8 may also determine spring growth habit. However, the presence of the dominant Vrn-A1d allele, which induces spring growth habit even as a single copy (Golovnina et al., 2010), in these accessions currently prevents definitive assessment of the Vrn-A1b.8 effect on spring type. The presence of two dominant alleles in one accession could result from plant material heterogeneity or copy number variation (CNV) due to locus duplication. Fixation of two different Vrn-1 alleles in T. dicoccoides genomes has been previously demonstrated (Konopatskaia et al., 2016). Moreover, this phenomenon has been observed in several other tetraploid wheat species (Golovnina et al., 2010; Chhuneja et al., 2015). Therefore, CNV in the genomes of these three spring T. dicoccoides accessions with two Vrn-A1 promoter copies appears to be a more probable explanation

Following the determination of nucleotide sequences of the Vrn-A1 gene promoter in this study, we evaluated the relationship between its allelic variants and the vegetation period duration in wild emmer. For our analysis, we supplemented the data with heading time values from several other studies where promoter allelic variants had been precisely identified through nucleotide sequencing (Shcherban et al., 2015b; Konopatskaia et al., 2016; Muterko et al., 2016). Combining data from different investigations, even under similar conditions, may introduce certain biases, although excluding portions of observations seemed unjustified as it would substantially reduce our accession sample (bulk). Considering the specific nature of our data, we selected the 2B-PLS method for statistical analysis due to its advantages over traditional approaches. Classical methods such as ANOVA and multiple regression require strict assumptions, including normal distribution, and demonstrate high sensitivity to multicollinearity and outliers. The use of quadratic criteria in these methods can lead to biased estimates, particularly with small sample sizes, increasing the probability of Type I errors. In contrast, the 2B-PLS method offers greater robustness through its use of latent variables, resulting in reduced sensitivity to outliers and multicollinearity. These characteristics make our chosen method particularly suitable for analyzing biological data characterized by statistical noise and complex factor interactions, which is especially important given our study’s specific features, including limited sample sizes.

Since copy number variation (CNV) of the dominant Vrn-A1 gene affects the duration from emergence to heading (Grogan et al., 2016), we considered the presence of two different alleles of dominant Vrn genes in a single accession as a distinct variant. We established correlation coefficients of r = 0.534 and r = 0.478 for the relationships “heading time × allelic variants” and “heading time × regions of origin”, respectively. While these coefficients allowed assessment of parameter relationships, their values preclude definitive conclusions about whether Vrn-A1 allelic variants or region-specific genetic factors predominantly influence the vegetation period duration trait in T. dicoccoides.Previous studies have repeatedly demonstrated significant effects of specific dominant Vrn-A1 alleles on maturation timing in common wheat (Royo et al., 2020; Qiu et al., 2021; Chumanova, Efremova, 2024). Our results suggest that analogous effects of this gene’s allelic variants in wild emmer T. dicoccoides are less pronounced. We acknowledge that our experiments were conducted exclusively under controlled greenhouse conditions without replicates and with a limited number of accessions. These methodological features impose certain limitations on result interpretation. Nevertheless, despite these limitations, our data demonstrate weak associations between the studied parameters. Similar experimental approaches – particularly testing under controlled conditions without replicates – have been employed in previously published studies evaluating plant heading times (Kippes et al., 2014, 2015; Palomino, Cabrera, 2023). Despite simplified designs, these authors confirmed phenotypic differences between compared groups, supporting the validity of such approaches. Additionally, we must acknowledge that our study analyzed only the Vrn-A1 promoter region. While this is the most variable region in T. dicoccoides, combinations of the Vrn-A1 with Vrn-B1 alleles, as well as Ppd-1 allele combinations, may influence vegetation period duration. Genotypes characteristic of specific wild emmer collection regions showed lower correlation with heading times than Vrn-A1 allelic variants, suggesting minimal influence of geographic origin on plant heading times.

The information obtained in this study could be valuable for breeding spring bread and durum wheats, particularly considering that T. dicoccoides is actively used as a genetic donor for these species (Badaeva et al., 2024). Furthermore, studies have demonstrated that Aegilops tauschii Coss. (syn. Ae. squarrosa L.) accessions (Takumi et al., 2011; Chepurnov et al., 2023), similar to T. dicoccoides (Table 1), exhibit significant polymorphism in the duration of the vegetation period trait. Therefore, hybridization of Ae. tauschii with spring T. dicoccoides accessions could facilitate the production of artificial hexaploid (2n = 6x = 42) amphidiploids, which may serve as a promising platform for successful introgression of novel dominant Vrn gene allelic variants determining vegetation period duration from these species into bread wheat.

## Conclusion

This study identified a novel Vrn-A1b.8 allele in spring T. dicoccoides accessions. We detected a significant association (p-value = 3.88 × 10–6) between allelic variants of the dominant Vrn-A1 gene and vegetation period duration, as well as an association (p-value = 4.92 × 10–5) between this parameter and the geographic origins (collection sites) of the wild emmer accessions. The research revealed that some T. dicoccoides accessions sharing identical Vrn-A1 alleles and originating from the same eco-geographical region show substantial variation in duration of the vegetation period. The observed phenotypic variability for this trait despite identical Vrn-A1 allelic composition suggests the involvement of additional genetic determinants controlling this characteristic in these accessions. These findings highlight the potential value of wild emmer accessions as genetic resources (donors) for expanding the genetic diversity of commercial bread and durum wheat varieties.

## Conflict of interest

The authors declare no conflict of interest.
